# Effect of American genomic ancestry on severe toxicities in children with acute lymphoblastic leukemia in the Amazon region

**DOI:** 10.1007/s12672-024-01014-z

**Published:** 2024-05-18

**Authors:** Alayde Vieira Wanderley, Francisco Cezar Aquino de Moraes, Giovanna Gilioli da Costa Nunes, Esdras Edgar Batista Pereira, Luciana Pereira Colares Leitão, Marcelo Braga de Oliveira, Ágatha Tereza Miranda Tavares, Laudreisa da Costa Pantoja, Bruna Cláudia Meireles Khayat, Marianne Rodrigues Fernandes, Paulo Pimentel de Assumpção, Ândrea Kely Ribeiro dos Santos, Rommel Mario Rodríguez Burbano, Sidney Emanuel Batista dos Santos, Raul Ribeiro, André Salim Khayat, Ney Pereira Carneiro dos Santos

**Affiliations:** 1 Otávio Lobo Children’s Cancer Hospital-Belém, Belém, PA 66063-240 Brazil; 2 Oncology Research Center, Federal University of Pará, University Hospital João de Barros de Barreto. , Rua Dos Mundurucus, no 4487, Belem, PA 66073-005 Brazil; 3 Laboratory of Human and Medical Genetics, Institute of Biological Science, Federal University of Pará, Belem, 66077-830 Brazil; 4 Ophir Loyola Hospital, Belém, PA 66063-240 Brazil; 5 St. Jude Children’s Research Hospital, Memphis, USA; 6 Afya Faculty of Medical Sciences of Palmas, Palmas 77.017-004, Tocantins, Brazil

## Abstract

**Background:**

Acute Lymphoblastic Leukemia (ALL) is a neoplasm of the hematopoietic system characterized by a clonal expansion of abnormal lymphocyte precursor cells. ALL is the most common form of cancer in children, but despite advances in treatment, it can still be fatal. Ethnic differences influence survival rates, and genomic ancestry plays an important role, especially in mixed-race populations such as Latin America. This study aims to analyze the influence of genomic ancestry on toxicity in children with ALL in the Amazon region.

**Methods:**

The study included 171 patients (protocol number 119,649/2012—Ethics Committee) with ALL treated at a pediatric treatment center in Belém do Pará, in the Brazilian Amazon. The patients were submitted to the BFM protocol of induction therapy for ALL. Toxicity was assessed based on laboratory tests and adverse events, classified according to the CTC-NCI guide. Genomic ancestry was determined using autosomal informative markers.

**Results:**

The majority of children (94.74%) developed some type of toxicity during treatment, 87.04% of which were severe. Infectious toxicity was the most common, present in 84.8% of cases, 77.24% of which were severe. Amerindian ancestry showed an association with the risk of severe general toxicity and severe infectious toxicity, with a contribution of 35.0% demonstrating a significant increase in risk. In addition, post-induction refractoriness and relapse were also associated with an increased risk of death.

**Conclusion:**

This study highlights the influence of Amerindian genomic ancestry on response to therapy and toxicity in children with ALL in the Amazon region. Understanding these associations can contribute to personalizing treatment and improving clinical outcomes.

**Supplementary Information:**

The online version contains supplementary material available at 10.1007/s12672-024-01014-z.

## Introduction

Acute Lymphoblastic Leukemia (ALL) is a hematopoietic neoplasm, defined as a clonal expansion of abnormal precursor lymphocytes (B and/or T cells), with complex and multifactorial events leading to malignant transformation, influenced by various factors [1, 2]. ALL is the most common form of cancer in children worldwide, and while therapeutic alternatives exist, it is still clinically manageable. However, ALL can lead to a fatal relapse, resulting in early mortality [3, 4].

Advancements in chemotherapy and knowledge about diagnostic alternatives for ALL have emerged in recent decades. The possibility of new drugs and therapeutic targets has led to the introduction of cellular therapies and the implementation of precision medicine to identify genomic markers that assist in treatment guidance and prognosis, significantly improving the survival rate of ALL. It has reached a cure and recovery rate of 90% in children living in developed countries, a reality not experienced in Latin American or low-income countries, where outcomes for children and adults are considerably worse than those in developed countries [4–8].

Ethnic differences in survival rates after childhood ALL vary worldwide [9–14], with Hispanic-origin children exhibiting poorer survival rates. Interestingly, Hispanic patients treated in high-income countries showed lower survival rates, highlighting ancestral genomic bias as a contributing factor to these outcomes. Particularly for the Latin American population, a highly heterogeneous group with genetic heritage contributions from European, African, and Native American ancestry [4, 15–17].

Taking these ancestral influences into account, studies conducted with more recent data in populations with a high Amazonian Amerindian ancestry have shown that 87% of ALL patients treated with the European Berlin-Frankfurt-Münster (BFM) Group protocol experienced grade 3 and 4 toxicities [16], a higher frequency than that found in other global populations subjected to the same protocol (26%) [18]. In addition to toxicity from standard ALL treatment, other studies in populations with high Native American ancestry showed a high risk of ALL recurrence [15].

Therefore, the different responses to medications related to ethnic differences can be partially explained by variations in the frequencies of important functional gene variants that make up the pharmacokinetic and pharmacodynamic pathways of chemotherapy agents, and these changes may be directly linked to ancestral genomic heritage. Considering these pieces of evidence in the literature, the present study aims to analyze the influence of genomic ancestry on the development of toxicity in pediatric patients diagnosed with ALL in the Amazon region.

## Methods

### Ethical considerations

The protocol used in this study was approved by the Research Ethics Committee of the Institute of Health Sciences of the Federal University of Pará, under protocol number 119,649/2012. Individuals under 18 years of age and their guardians were duly informed about the research. Volunteers agreed to participate in the study by signing the Informed Consent Form, and their guardians also signed the Informed Consent Form. Our methodology has already been described by Da Silva et al. [19]

### Study populations

This is a prospective study that included 171 patients newly diagnosed with ALL by immunophenotyping and treated at a reference center for pediatric oncology (Otávio Lobo Hospital) in Belém do Pará, in the Brazilian Amazon. The included patients were between 1 and 18 years old, had no recurrence, comorbidities, or other types of cancer, and had morphological, immunophenotypic, and, when available, molecular diagnoses. We excluded patients with Down's syndrome, sickle cell anemia, HIV, tuberculosis, hepatitis B or C, who had previously undergone radiotherapy or chemotherapy for any type of cancer and who had previously undergone hematopoietic stem cell transplantation.

### LLA induction therapy protocol

The included patients underwent induction therapy for ALL with the BFM 2009 protocol. During the induction therapy, which lasts a total of 64 days, patients receive phase 1 corticosteroids for 36 days; four doses of vincristine; 2 doses of doxorubicin (in low and intermediate-risk patients) or 4 doses of doxorubicin (in high-risk patients); and l-asparaginase (8 doses). During the second phase of induction with cyclophosphamide (two doses), patients received 16 doses of cytarabine and 28 consecutive days of mercaptopurine. Supplementary Table 1 summarizes the details of the treatment protocol used during this study.

### Toxicity evaluation and classification

Laboratory tests, including transaminases and complete blood counts, were recorded in a table at three time points during the induction phase: on the first day of treatment before using any medication, before the second phase of induction before using mercaptopurine but with patients already sensitized to other drugs, and after the second phase of induction. Adverse events such as anorexia, colitis, diarrhea, dyspepsia, mucositis, nausea, vomiting, neutropenia, and documented or undocumented infection were recorded, and numerical stratification was applied to the events according to the CTC-NCI (Common Toxicity Criteria of the US National Cancer Institute). After collecting information, adverse effects were classified by grade, considering CTC-NCI: mild/moderate (grades 0, 1, and 2) and severe (grades 3 and 4).

### DNA extraction and quantification

DNA was extracted using the conventional phenol–chloroform method according to Sambrook. Samples were quantified using the NanoDrop ND-1000 equipment (Thermo Scientific NanoDrop Products, Wilmington, DE, USA).

### Genomic ancestry

The analysis was performed using a panel of 61 informative autosomal ancestry markers according to [23]. Two multiplex PCRs were performed, followed by electrophoresis on the ABI Prism 3130 sequencer (Applied Biosystems, Life Technologies, Carlsbad, CA, USA) and analysis using the GeneMapper ID v.3.2 software (Applied Biosystems, Life Technologies, Carlsbad, CA, USA). Individual proportions of European, African, and Amerindian genetic ancestry were estimated using Structure software v.2.3.3.

### Statistical analysis

A descriptive analysis of the data related to the sample characterization was performed. Quantitative variables were first subjected to the Kolmogorov–Smirnov test to analyze the normality distribution. The protocol for analysis of this study has been reported by Da Silva et al. [19].

For comparative analysis between the study groups regarding sample characterization variables, the chi-square test was applied for categorical variables, and the Mann–Whitney test was used for continuous variables. To analyze the association of variables with the risk of overall severe toxicity, severe infectious toxicity, and the occurrence of deaths in the treatment of children with ALL, simple logistic regression was performed.

All statistical analyses were conducted using the SPSS 20.0 (IBM, Stanford University, USA) statistical package, respecting the significance level of 5% (p ≤ 0.05).

## Results

**Fig. 1:**
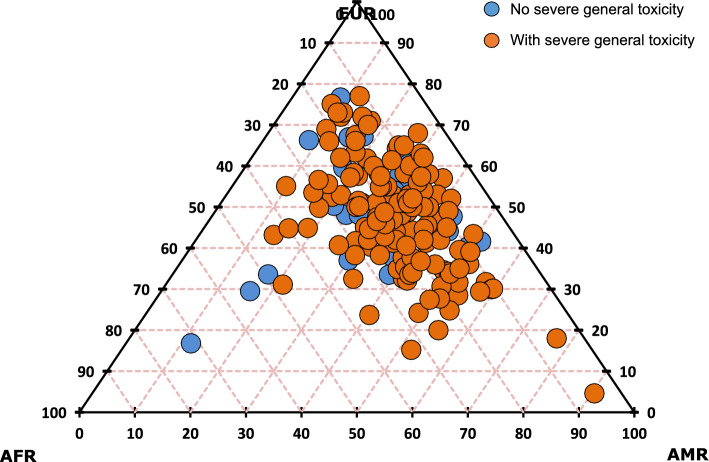
Genomic ancestry of children with Acute Lymphoid Leukemia, due to the development of severe toxicity. The proximity at each apex of the triangle represents how positively correlated ancestry is with the development of toxicity. *EUR* European, *AFR* African, *AMR* Amerindian

In the present study, it can be observed that 94.74% (95% CI 91.2–97.7) of children with ALL developed some type of toxicity during treatment, among these, 87.04% (95% CI 82.1–92.0) presented severe toxicity. Among the toxicities, the most prevalent was infectious toxicity, present in 84.8% (95% CI 78.9–90.1) of cases, and of these, 77.24% (95% CI 70.3–84.8) developed severe infectious toxicity, as shown in Table 1. Furthermore, it can be observed that the children with ALL included had predominantly European genomic ancestry (47.5%), followed by Amerindian (32.6%) and African (19.5%) (Table 1 and Fig. 1).

**Table 1:** Analysis of the occurrence of general and specific toxicity and its degrees, and the genomic ancestry of children with Acute Lymphoid Leukemia in the Amazon Region

Variables	Frequency	Percentage	CI95%
General toxicity			
Yes	162	94.74	91.2–97.7
No	9	5.26	2.3–8.8
Degrees of general toxicity			
Moderate	20	12.35	7.4–17.3
Severe	141	87.04	82.1–92.0
Types of toxicity			
Infectious	145	84.80	78.9–90.1
Hematological	100	58.48	50.9–66.1
Gastrointestinal	115	67.25	59.6–73.7
Neurological	29	16.96	11.7–22.2
Infectious toxicity			
Moderate	30	20.69	13.8–27.6
Severe	112	77.24	70.3–84.8
Hematological toxicity			
Moderate	41	41.00	31.3–51.5
Severe	58	58.00	48.5–67.7
Gastrointestinal toxicity			
Moderate	38	33.04	24.4–41.7
Severe	75	65.22	56.5–73.9
Neurological toxicity			
Moderate	16	55.17	34.5–72.4
Severe	13	44.83	27.6–65.5
Ancestry			
European	0.475 (± 0.131)		0.456–0.495
Amerindian	0.326 (± 0.132)		0.307–0.347
African	0.195 (± 0.084)		0.183–0.203

*CI* Confidence interval

**Fig. 2:**
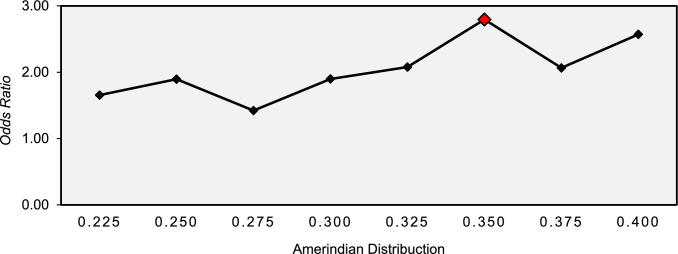
Variation in the odds ratios recorded for different distributions of genomic ancestry for severe general toxicity in patients with ALL in the Amazon Region

When assessing the risk factors associated with the occurrence of severe general toxicity, the contribution of Amerindian genomic ancestry was greater among children who developed severe general toxicity when compared to those who did not (OR 28.076; 95% CI 1.007–782.92). In addition, Amerindian ancestry was significantly associated with an increased risk of severe general toxicity (OR: 28.076; p-value: 0.049), as shown in Table 2 and Fig. 2.

**Table 2:** Analysis of factors associated with the occurrence of severe general toxicity in children with Acute Lymphoid Leukemia in the Amazon Region

Variables	Severe toxicity		p-value^a^	OR (IC95%)
	Yes (n = 141)	No (n = 30)		
Sex				
Male	89 (63.1%)	19 (63.3%)	0.982	0.991 (0.437–2.244)
Female	52 (6.9%)	11 (36.7%)		
Age (years)				
Mean (± SD)	5.00 (± 2.97)	6.19 (± 3.92)	0.122	1.098 (0.975–1.237)
Laboratory				
Initial hemoglobin	7.87 (± 2.11)	7.75 (± 2.45)	0.809	0.979 (0.826–1.161)
Initial leukometry	39702 (± 109563)	56056 (± 99497)	0.460	1.000 (1.000–1.000)
Initial platelets	68816 (± 86909)	70936 (± 90705)	0.906	1.000 (1.000–1.000)
Ancestry				
European	0.472 (± 0.133)	0.488 (± 0.124)	0.595	0.535 (0.053–5.366)
Amerindian	0.336 (± 0.135)	0.283 (± 0.117)	0.049*	28.076 (1.007–782.92)
African	0.192 (± 0.078)	0.217 (± 0.107)	0.271	0.082 (0.001–6.992)

*SD* Standard deviation, *OR* Oddis ratio, *CI* Confidence interval

^a^Simple logistic regression

^*^p < 0.05

The influence of the distribution of Amerindian ancestry on the susceptibility to developing severe general toxicity in children with ALL was assessed using a logistic regression model by contribution ranges (Fig. 2). The analysis showed that Amerindian ancestry in the range between 22.5% and 40.0% showed an increased risk for this event. However, only a contribution of 35.0% showed a significant increase in risk (OR:2.795; 95% CI 1.113–10.077; p-value: 0.035). The other genomic ancestries showed no significant associations.

**Fig. 3:**
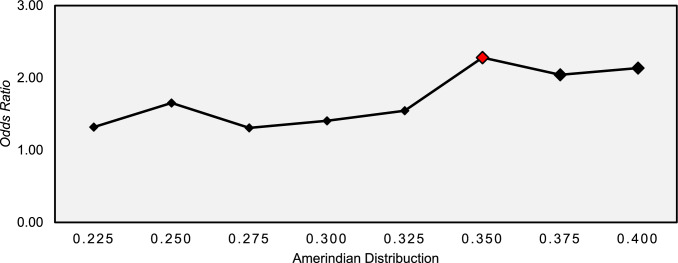
Variation in the odds ratios recorded for different distributions of genomic ancestry for severe infectious toxicity in patients with ALL in the Amazon Region

In assessing the risk factors associated with the occurrence of severe infectious toxicity, it can be seen that the contribution of Amerindian genomic ancestry was greater among children who developed severe infectious toxicity when compared to those who did not. In addition, Amerindian ancestry was significantly associated with an increased risk of severe infectious toxicity (OR: 13.689; 95% CI 1.050–178.65; p = 0.046), as can be seen in Table 3 and Fig. 3.

**Table 3:** Analysis of factors associated with the occurrence of severe infectious toxicity in Acute Lymphoid Leukemia children in the Amazon Region

Variables	Severe toxicity		p-value^a^	OR (IC95%)
	Yes (n = 112)	No (n = 59)		
Sex				
Male	72 (64.3%)	36 (61.0%)	0.674	1.150 (0.600–2.204)
Female	40 (35.7%)	23 (39.0%)		
Age (years)				
Mean (± SD)	6.12 (± 4.02)	5.73 (± 3.33)	0.525	1.028 (0.944–1.119)
Laboratory				
Initial hemoglobin	7.76 (± 2.59)	7.80 (± 1.98)	0.903	0.992 (0.868–1.133)
Initial leukometry	60193 (± 102525)	39887 (± 98085)	0.221	1.000 (1.000–1.000)
Initial platelets	72401 (± 93767)	67076 (± 82415)	0.712	1.000 (1.000–1.000)
Ancestry				
European	0.467 (± 0.138)	0.489 (± 0.121)	0.307	0.281 (0.025–3.212)
Amerindian	0.341 (± 0.140)	0.298 (± 0.113)	0.046*	13.689 (1.050–178.65)
African	0.191 (± 0.080)	0.204 (± 0.091)	0.316	0.149 (0.004–6.142)

*SD* Standard deviation, *OR* Oddis ratio, *CI* Confidence interval

^a^Simple logistic regression

^*^p < 0.05

The influence of the distribution of Amerindian ancestry on susceptibility to the development of severe infectious toxicity in children with ALL was assessed using a logistic regression model by contribution ranges (Fig. 3). The analysis showed that Amerindian ancestry in the range between 22.5% and 40.0% contribuition of Amerindian genetic profile showed an increased risk of this event. However, only a contribution of 35.0% showed a significant increase in risk (OR: 2.281; 95% CI 1.182–4.732; p-value: 0.020). The other genomic ancestries showed no significant associations.

When evaluating the risk factors associated with the occurrence of deaths, it can be seen that post-induction refractoriness was higher among patients who died, significantly increasing the risk of death (p < 0.001; OR: 19.198). The same was observed for relapse, with a higher prevalence among children with ALL who died, significantly increasing the risk of death (p < 0.001; OR: 28.420). In addition, the occurrence of severe general toxicity (p-value: 0.012; OR: 3.421) and severe infectious toxicity (p-value: 0.002; OR: 2.933) were also associated with an increased risk of death among children undergoing treatment for ALL, as detailed in Table 2 in the Supplementary Material.

## Discussion

Genomic studies are still very rare in populations of Amerindian origin and populations mixed with them. Different studies in the world literature have shown a worse prognosis for Acute Lymphoid Leukemia and high toxicities associated with standard therapy—BFM protocol—for the disease in populations with high Amerindian ancestry [8].

Therefore, it is important to highlight that racial and ethnic differences are significant in the prognosis of patients, since children of populations with Amerindian ancestry have, in addition to a worse treatment outcome, a higher incidence of ALL [20–22].

The results of this study shows that the population in the Brazilian Amazon, characterized by a high degree of miscegenation with Amerindians (around 30%), has proportionally high general (94.7%) and severe (87.04%) toxicities [23]. These data show significant differences from the worldwide data on the standard protocol used in ALL therapy.

In our patient sample undergoing therapy for ALL in the Brazilian Amazon, it was possible to identify approximately 10 indigenous individuals with lethal toxicities to the treatment. Therefore, based on this impactful result, the study's objective is to investigate the correlation between the genomic ancestry of these populations and the respective toxicity data.

The results based on the average ancestry in the patient group show similarity to the results obtained from the same population in northern Brazil [23]. Specifically, in Indigenous genomic ancestry, we observed results of 30,6 contribuition of Amerindian genetic profile in the population comprising the patient sample and 30.95% in the group of patients with ALL. When we separately analyzed individuals with severe and non-severe toxicity, we found a significant difference in our results for Amerindian ancestry. This result reinforces studies that suggest Amerindian ancestry may be a risk factor for toxicity and a worse prognosis [24–27].

When correlating ancestries with toxicities, we found interesting results that indicate severe and infectious toxicity were positively associated with Amerindian ancestry starting from 35%. When quantifying the influence of Amerindian ancestry in the study, it becomes apparent that patients with 35% or more contribuition of Amerindian genetic profile exhibited a significantly elevated risk for the development of toxicity. Therefore, we can assert that, concerning ALL patients, individuals with ancestry above 35% Amerindian have a higher risk of experiencing toxicity with the use of standard ALL therapy. This result may be supported by other studies in the global literature that highlight this elevated risk in individuals of mixed Amerindian heritage and traditional Amazonian indigenous populations [21–24].

Our hypothesis regarding these results leads us to the idea that there is limited research on the genomics of Amerindians, particularly Amerindian pharmacogenetics and mixed populations. Thus, the correlation of Amerindian ancestry with the severe toxicities observed may potentially result from specific gene variants within this ethnic group found in mixed populations. Other studies investigating the exomes of indigenous people have already demonstrated the existence of novel variants in other genes important for pharmacogenetics, which further supports our hypothesis [29–31].

Numerous studies have illustrated that Native American populations exhibit a distinct pattern of variability within the immune system. With regard to our finding of a correlation between Amerindian ancestry and severe infectious toxicity, we can explain it by the fact that Amazonian Amerindians have, throughout their generations, intermarried and have undergone drastic population reductions, which has a direct impact on the diversity of the immune response [26–28].

The Brazilian population is the largest admixture in the world, and this has a direct impact on the variation of important genetic variants related to pharmacogenomics. In this way, the international protocols that recommend the use of drugs do not consider the fluctuation of these variants in the Brazilian population, or even the presence of new variants that are specific to Brazilian admixture populations. Knowledge of these very poorly studied mixed-race populations in Latin America could directly lead to the reformulation of new precision medicine protocols for therapies that can be used in these populations, taking into account, in this case, the fluctuation in the allele frequencies of important variants already identified by international agencies and new variants that have not yet been identified.

## Conclusion

Our work reinforces the fact that mixed-race and indigenous Amerindian populations have a higher risk of toxicity and a worse prognosis for treatment with the protocols used for ALL therapy. Therefore, the data presented is of great value for understanding the variability of response, and may provide new insights for the investigation and genomic analysis of genetic variants that may be directly related to greater severity of therapy for ALL.

### Supplementary Information

Supplementary material 1: Table S1. online provides the Analysis of the Prevalence of Toxicities by Treatment Phase in Children with Acute Lymphoblastic Leukemia in the Amazon Region. GTI Gastrointestinal. Table S2. online provides the Analysis of factors associated with mortality in children with Acute Lymphoblastic Leukemia in the Amazon Region. Figure S1. online provides the Genetic Ancestry and Susceptibility to Severe General Toxicity in Children with Acute Lymphoblastic Leukemia in the Amazon Region. Case-Toxicity; Control; without toxicity. Figure S2. online provides the **. **Genetic Ancestry and Susceptibility to Severe Infectious Toxicity in Children with Acute Lymphoid Leukemia in the Amazon Region.

## Data Availability

All data generated or analysed during this study are included in this published article.
