# Supplementary material for: Effect of American genomic ancestry on severe toxicities in children with acute lymphoblastic leukemia in the Amazon region
Source: Discov Oncol. 2024 May 18;15:171. doi: 10.1007/s12672-024-01014-z (PMC11102409; doi:10.1007/s12672-024-01014-z)
Supplement: Supplementary file 1 — Supplementary material 1: Table S1. online provides the Analysis of the Prevalence of Toxicities by Treatment Phase in Children with Acute Lymphoblastic Leukemia in the Amazon Region. GTI Gastrointestinal. Table S2. online provides the Analysis of factors associated with mortality in children with Acute Lymphoblastic Leukemia in the Amazon Region. Figure S1. online provides the Genetic Ancestry and Susceptibility to Severe General Toxicity in Children with Acute Lymphoblastic Leukemia in the Amazon Region. Case-Toxicity; Control; without toxicity. Figure S2. online provides the . Genetic Ancestry and Susceptibility to Severe Infectious Toxicity in Children with Acute Lymphoid Leukemia in the Amazon Region. [file 12672_2024_1014_MOESM1_ESM.docx]

#
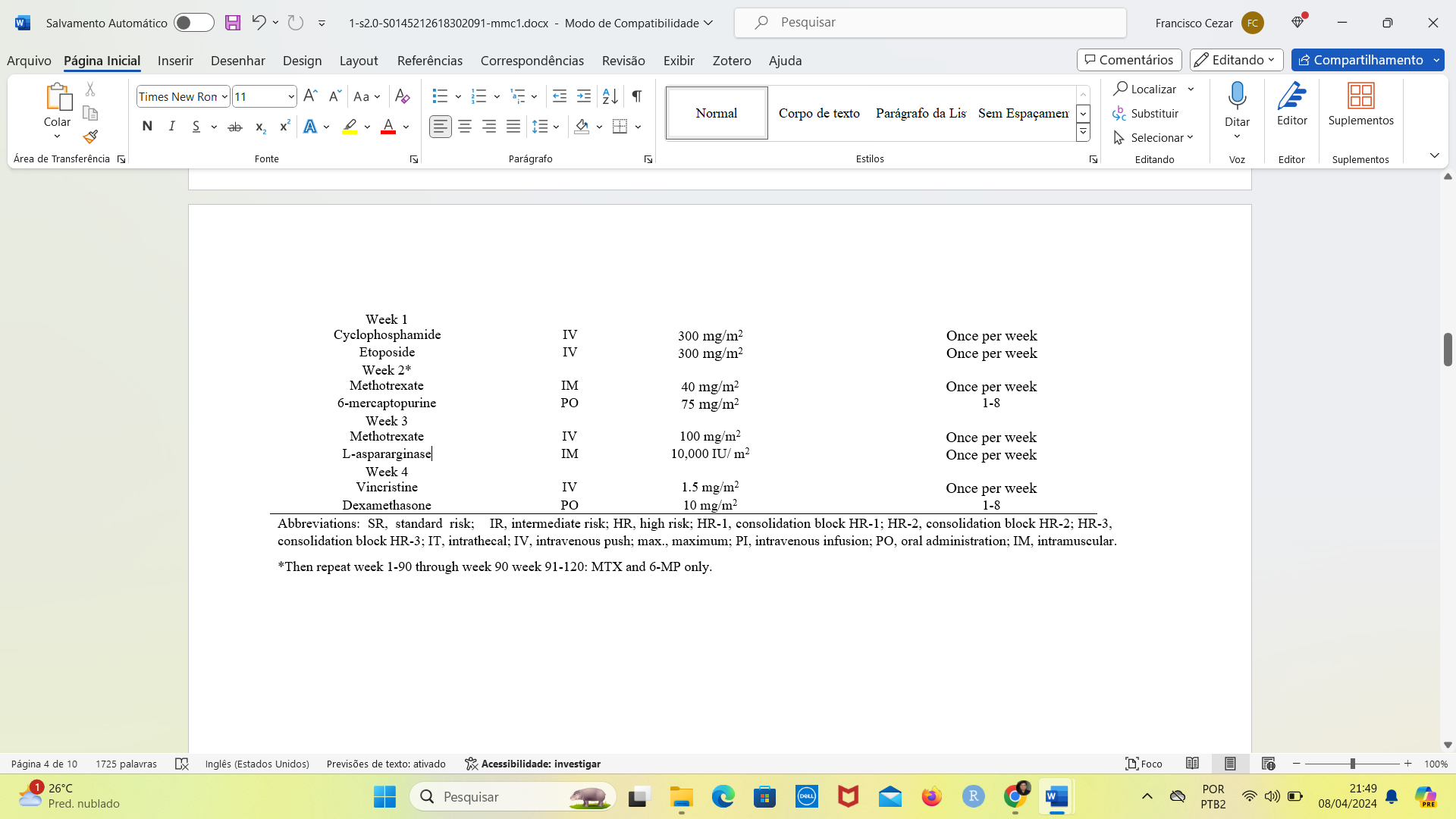

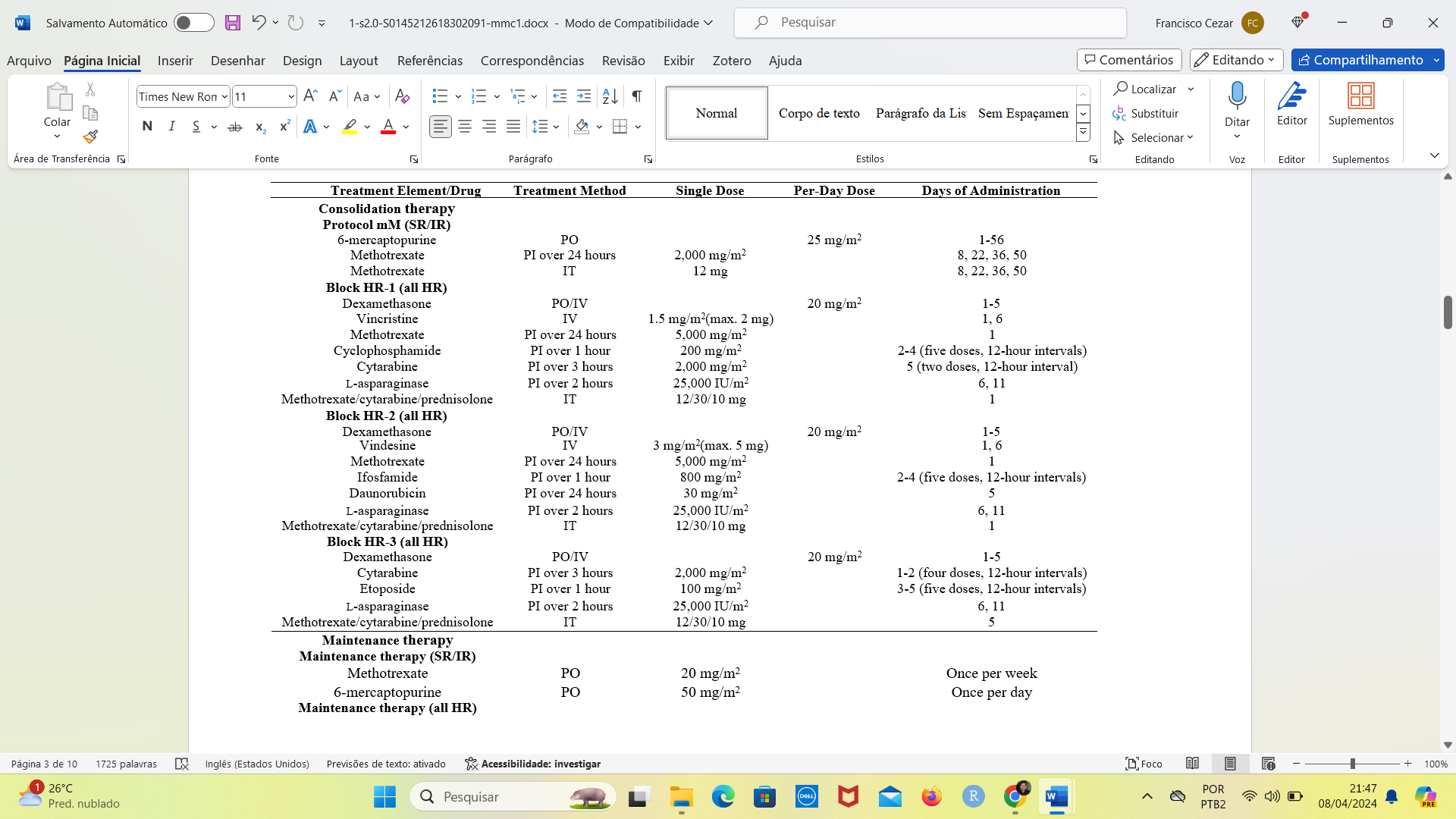
Table 1. Treatment

| **Toxicidades** | **Phase I** | **Phase II** | **Phase III** | **Phase IV** | **p-value^a^** |
| --- | --- | --- | --- | --- | --- |
| Infectious Toxicity | 89 (44.7) | 51 (41.5) | 75 (41.7) | 33 (33.0) | 0.0043* |
| Neurological Toxicity | 5 (2.5) | 16 (13.0) | 9 (5.0) | 1 (1.0) |  |
| GTI Toxicity | 53 (26.6) | 29 (23.6) | 47 (26.1) | 37 (37.0) |  |
| Hematological toxicity | 52 (26.1) | 27 (22.0) | 49 (27.2) | 29 (29.0) |  |

**Table 2.** Analysis of the prevalence of toxicities by treatment phase in children with Acute Lymphoblastic Leukemia in the Amazon Region. GTI. Gastrointestinal.


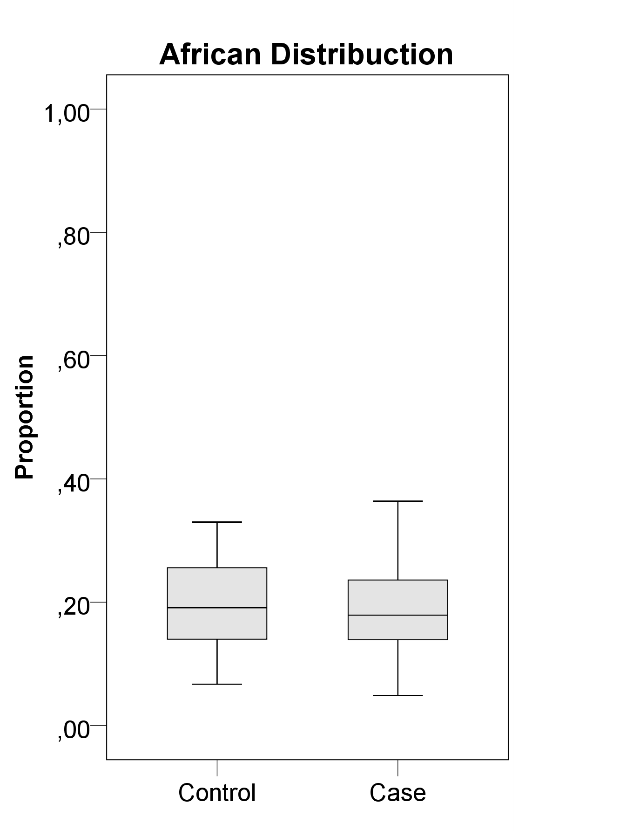

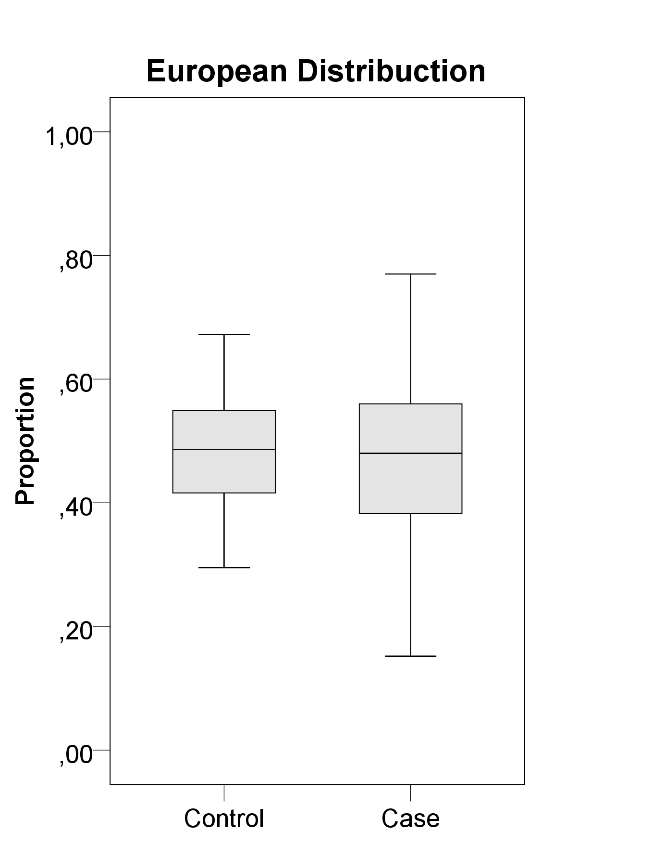

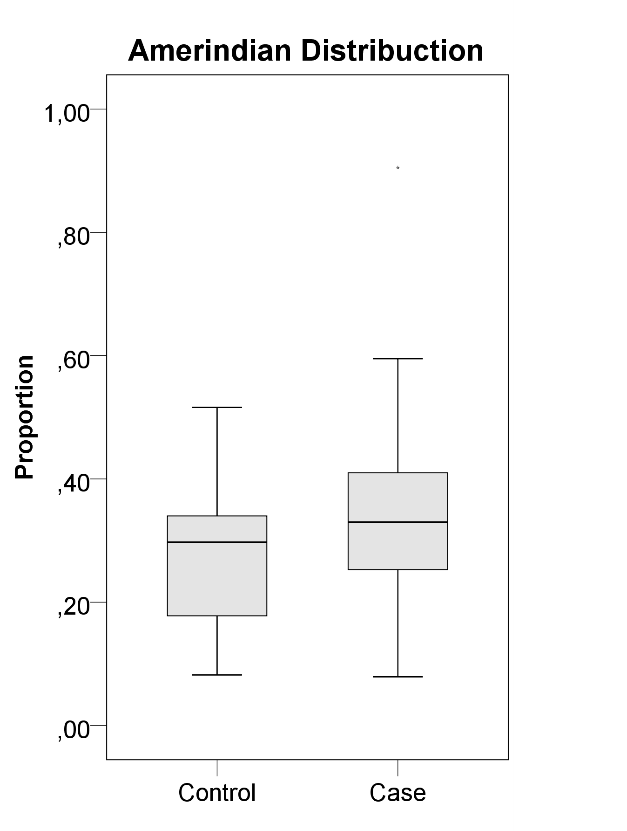


p=0.595

p=0.049*

p=0.271

**Figure 1.** Genetic ancestry and susceptibility to severe general toxicity in children with Acute Lymphoid Leukemia in the Amazon Region. Case-Toxicity; Control; no toxicity.


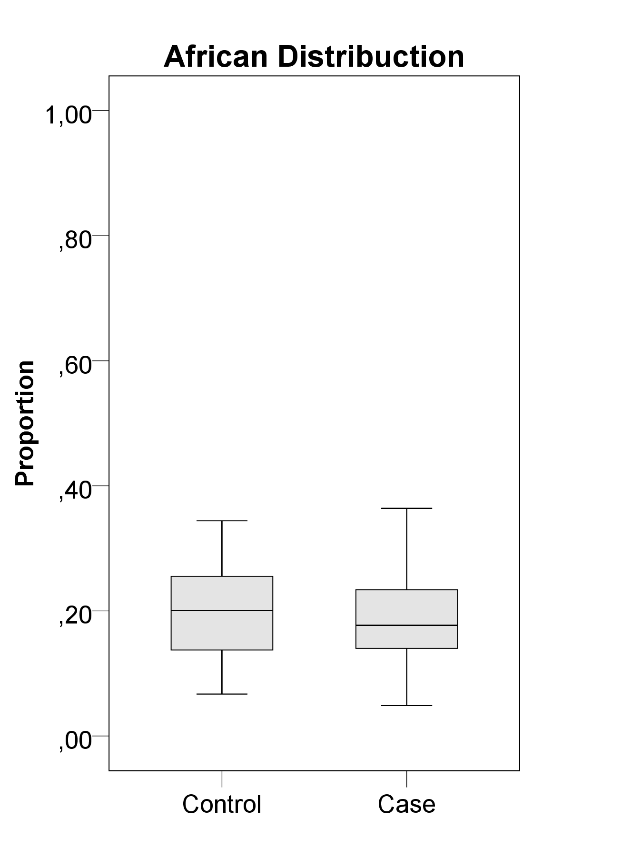

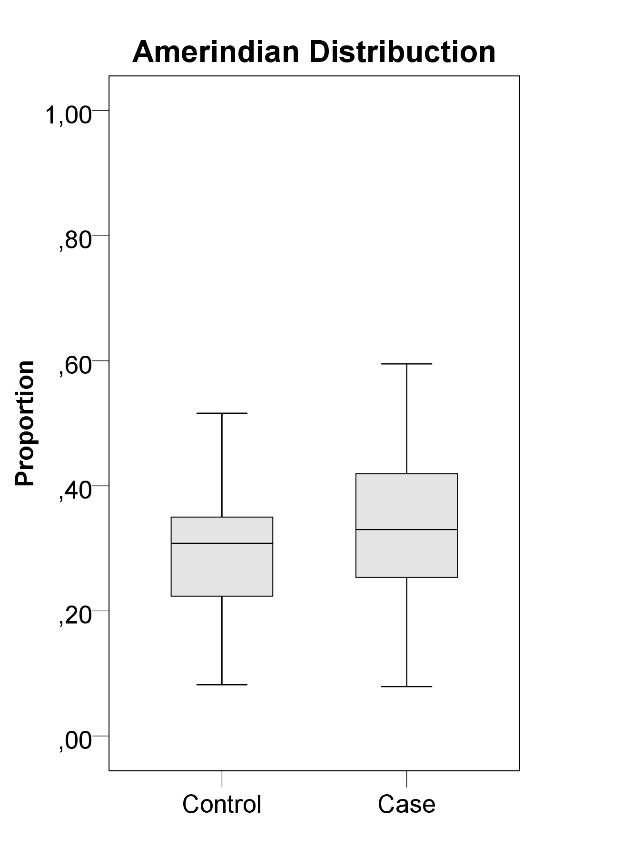

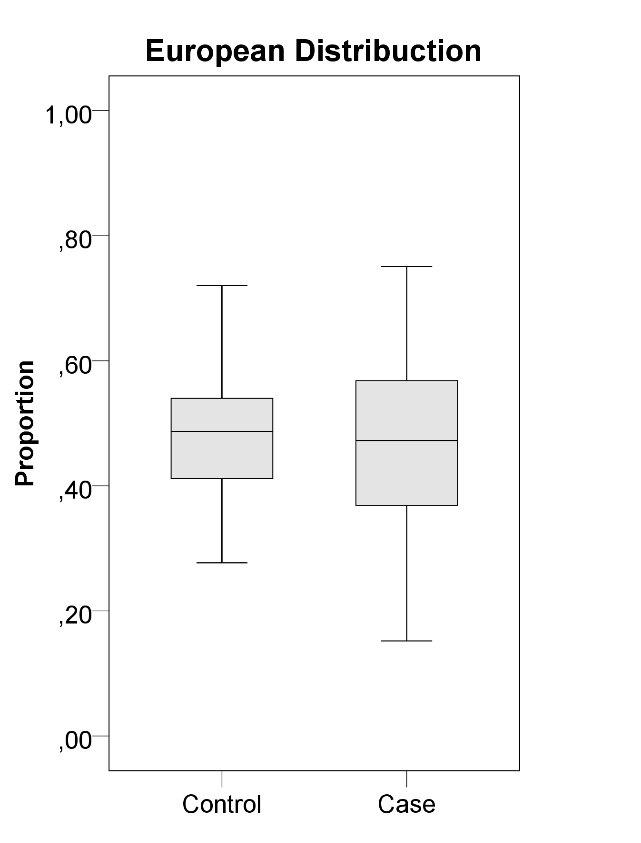


p=0.307

p=0.046*

p=0.316

**Figure 2.** Genetic ancestry and susceptibility to severe infectious toxicity in children with Acute Lymphoid Leukemia in the Amazon Region.

**Table 3.** Analysis of factors associated with the occurrence of deaths in children with Acute Lymphoid Leukemia in the Amazon Region.

| **Variables** | **Death** | | **p-value^a^** | **OR (IC95%)** |
| --- | --- | --- | --- | --- |
|  | **Yes (n=71)** | **No (n=100)** |  |  |
| **Gender** |  |  |  |  |
| Male | 47 (66.2%) | 61 (61.0%) | 0.488 | 1.252 (0.663 - 2.363) |
| Female | 24 (33.8%) | 39 (39.0%) |  |  |
| **Age (years)** |  |  |  |  |
| Mean (±dp) | 6.24 (±4.30) | 5.80 (±3.40) | 0.455 | 1.031 (0.952 - 1.117) |
| **Laboratory** |  |  |  |  |
| Initial Hemoglobin | 7.49 (±2.74) | 7.98 (±2.10) | 0.198 | 0.916 (0.802 - 1.047) |
| Initial leukometry | 62537 (±102545) | 46548 (±100201) | 0.313 | 1.000 (1.000 - 1.000) |
| Initial platelets | 59178 (±75092) | 78648 (±98500) | 0.170 | 1.000 (1.000 - 1.000) |
| **Post-induction outcome (D33)** | |  |  |  |
| Remission | 48 (71.6%) | 97 (97.9%) | **<0.001*** | 19.198 (4.29 – 85.85) |
| Refractoriness | 19 (28.4%) | 2 (2.1%) |  |  |
| Recurrence |  |  |  |  |
| No | 19 (28.4%) | 90 (91.8%) | **<0.001*** | 28.420 (11.58–69.73) |
| Yes | 48 (71.6%) | 08 (8.16%) |  |  |
| **Ancestry** |  |  |  |  |
| European | 0.476 (±0.133) | 0.474 (±0.131) | 0.897 | 1.166 (0.115 -11.857) |
| Amerindian | 0.318 (±0.125) | 0.332 (±0.138) | 0.492 | 0.443 (0.043 - 4.514) |
| African | 0.204 (±0.090) | 0.189 (±0.079) | 0.253 | 8.367 (0.220 - 18.34) |
| **Toxicity** |  |  |  |  |
| General Severe | 65 (91.5%) | 76 (76.0%) | **0.012*** | 3.421 (1.318 - 8.881) |
| Severe Hematological | 26 (36.6%) | 32 (32.0%) | 0.530 | 1.228 (0.647 - 2.329) |
| Severe Infectious | 56 (78.9%) | 56 (56.0%) | **0.002*** | 2.933 (1.467 - 5.867) |
| Severe Gastrointestinal | 35 (49.3%) | 40 (40.0%) | 0.228 | 1.458 (0.790 - 2.694) |
| Severe Neurological | 7 (9.9%) | 6 (6.0%) | 0.353 | 1.714 (0.554 - 5.335) |

dp. Standard Deviation. OR. Odds Ratio. CI. Confidence Interval. a. Simple Logistic Regression. *. p-value < 0.05.
